# An empirical assessment of a modified artificially intelligent device use acceptance model—From the task-oriented perspective

**DOI:** 10.3389/fpsyg.2022.975307

**Published:** 2022-08-09

**Authors:** Yutao Yang, Jia Luo, Tian Lan

**Affiliations:** ^1^Business School, Sichuan University, Chengdu, China; ^2^Business School, Chengdu University, Chengdu, China

**Keywords:** artificial intelligence, task-oriented AI device, technology acceptance, utilitarian motivation, task-technology fit, switching intention

## Abstract

Artificial intelligence (AI) is a cutting-edge technology that has been widely applied in tourism operations. To enhance tourists' experience, many tourism suppliers introduced AI devices to interact with tourists. Previous studies classified AI devices as task- and social- oriented based on their functions; however, current models that explain customers' intention to use AI devices did not reflect the discrepancy between the two different types. Therefore, this paper attempts to fill this gap by proposing a theoretical model for the use of task-oriented AI devices. Based on the multi-stage appraisal framework and the Structural Equation Modeling analysis, this paper presents the following findings: (1) utilitarian motivation, interaction convenience, and task-technology fit are the factors appraised in the first stage; (2) perceived competence and flow experience are the factors appraised in the second stage; (3) utilitarian motivation, interaction convenience, and task-technology fit are positively associated with perceived competence. (4) Perceived competence positively influences flow experience, which further affects customers' switching intention from task-oriented AI devices to human service; (5) the serial mediating effect of perceived competence and flow experience between the stimulus mentioned in the first appraisal stage and the switching intention is confirmed. This study reveals the underlying psychological mechanism when customers use task-oriented AI devices, and it provides a theoretical framework for task-oriented AI device adoption.

## Introduction

Tourism growth is now being accompanied by some new trends, such as AI technology, virtual reality, and the sharing economy. Recently, an increasing number of tourism suppliers have benefited from implementing intelligent automation to deliver enhanced customer experiences (Anurag, 2018). For instance, Spencer, an android robot, was introduced at Amsterdam Airport to guide passengers; Care-E, a self-driving trolley, was introduced at the airport to help flight passengers to carry their luggage and guide them to any point of interest; Xiaoyou, an e-commerce customer service bot was adopted to help customers with their itineraries. With the advancement of AI technology, the operators can serve customers more efficiently.

Although interacting with AI machines seems like a trend that cannot be avoided, not all customers are ready to accept it. As the findings of Lommatzsch (2018) showed, customers still preferred the human workforce when an emergency or complex issues occurred, since many AI machines can only perform limited functions, such as answering simple questions or performing designed actions. The argument was supported by Castelo et al. (2019). They found that consumers do not want to rely on algorithms to perform tasks that are usually done by humans, even though algorithms often outperform humans in those jobs (Castelo et al., 2019). Also, consumers rely less on algorithms if they find algorithms make mistakes (Dietvorst et al., 2018). For instance, consumers are more willing to choose human doctors over AI doctors because they think AI doctors are more likely to ignore individual uniqueness (Longoni et al., 2019). In the context of tourism and hospitality, AI service failures are complained about by tourists since they cannot handle complex issues flexibly due to the pre-set programs (Diskin, 2019; Tussyadiah et al., 2020; Lv et al., 2022). More specifically, some of the examples are: a guide robot cannot direct visitors to a destination that does not exist in its database, or an in-room AI assistant cannot correctly identify the commands of a guest (Diskin, 2019). Therefore, although a great number of service providers recommend tourists to use AI devices as default, customers may switch to human staff ultimately.

Previous literature mainly studied the antecedents of customers' intention to use AI devices on the basis of the existing technology acceptance theories, including the Technology Acceptance Model (TAM) (Sundar et al., 2016), Unified Theory of Acceptance and Use of Technology (UTAUT) (Fritz et al., 2016), and Artificially Intelligent Device Use Acceptance (AIDUA) model (Gursoy et al., 2019). However, except for the AIDUA model, the traditional technology acceptance models were originally developed for the utilization of non-intelligent technologies, and the features of AI technology were overlooked. As Lu et al. (2019) pointed out, the ease-of-use should not be included in previous models since AI devices did not require customers to learn how to operate them due to the humanlike intelligence they possess. Therefore, based on the cognitive Appraisal theory and cognitive dissonance theory, the AIUDA model was proposed to explain the process by that customers adopt AI devices during service counters.

Different technology acceptance models were examined in various contexts, such as airline service (West et al., 2018), hotel service (Tavakoli and Mura, 2018), and health care service (Hung, 2021); however, most of them were comprehensive models that did not differentiate between AI types. In fact, customers may treat different types of AI devices differently, which may be a different psychological path-way that has not been delineated. Samala et al. (2020) mentioned that the interaction forms between tourists and AI devices might be different in tourism services. For instance, the information an AI device provides to customers can be in the form of interactive messages, audio tours, interactive booking process, facial recognition technologies, chatbots, self-service technologies, language translations, etc. (Samala et al., 2020). Therefore, some researchers proposed that the types of AI devices can be classified into social- and task-oriented. Social-oriented AI devices refer to the ones that use informal and relational dialogs, namely small talk, emotional support, and customary greetings, to interact with customers by achieving social-emotional goals (Chattaraman et al., 2019; Lv et al., 2020, 2022). Examples include, but are not limited to Tess, a psychological AI (Fulmer et al., 2018), and ElliQ, a voice-operated care companion. Van et al. (2020) found companion robots can help tourists to reduce stress and loneliness during the COVID-19 pandemic by conducting social association. Task-oriented AI devices, on the other hand, are the ones that use more formal and purely on-task dialogs to serve the customers by achieving functional goals (Chattaraman et al., 2019). For example, Pepper, a guide robot, was introduced at a tourist destination to complete the task of guiding tourists around (Go et al., 2020). The Facebook Messenger robot, launched by the world's leading online travel agency Expedia, was utilized to assist tourists in the booking process (Popesku, 2019). According to the cognitive load theory, performance might be reduced when customers divide their attention between information sources and process information that is peripheral (Veletsianos, 2012). Therefore, when customers adopt AI devices for different purposes, the discrepancy might exist in the antecedents that lead customers to choose between task- and social-oriented AI devices. However, most current studies focused on social-oriented AI devices (Yang et al., 2017), and little attention was paid to task-oriented AI devices and the factors that influence the switching intention of customers from AI devices to human force.

Thus, to fill this gap, this study aims to propose a theoretical behavioral model toward tourists' use of task-oriented AI devices, which is named the Task-oriented AI Acceptance (T-AIA) model. More specifically, the goals of this paper are: (i) identifying the key antecedents of customers' switching intention from task-oriented AI devices to human service; (ii) delineating the underlying psychological mechanism.

## Theoretical background and hypotheses development

### Acceptance of AI devices

Recently, the number of research on the predictors and underlying mechanisms of technology acceptance for customers kept increasing (Kulviwat et al., 2007). Based on TRA and TPB theories, the technology acceptance model (TAM) was constructed to examine customers' willingness to accept a new technology (Davis et al., 1989). For instance, hedonic motivation is identified as a primary factor that influences the intention of customers to use AI devices (Niemelä et al., 2017). However, it is inadequate to use those models to study customers' acceptance of AI devices (Gursoy et al., 2019) since some core drivers (e.g., perceived usefulness) are more applicable for new technology learning (Lu et al., 2019).

On the basis of TAM, the unified theory of acceptance and use of theory (UTAUT) proposed four key predictors (e.g., utilitarian performance expectancy, effort expectancy, social influence, and facilitating conditions) that influence the users' behavioral intention to use a technology (Venkatesh et al., 2003), which is an extension model. However, UTAUT mainly focused on the adoption of non-intelligent technology (Mortenson and Vidgen, 2016; Lu et al., 2019), such as mobile check-in, self-service kiosks, and e-bank.

Although TAM and UTAUT models were introduced in many research to study the acceptance of different kinds of AI devices (Table 1), such as voice assistants (Moriuchi, 2019; Pal et al., 2020), smart wearable devices (Park, 2020), and conversational AI (Vimalkumar et al., 2021), TAM and UTAUT models did not capture the multi-faceted role of AI devices, which was pointed out by Gursoy et al. (2019). The comprehensive theoretical model of AI device use acceptance was proposed by Gursoy et al. (2019) based on the cognitive appraisal theory. The model holds that there are six key predictors (social influence, hedonic motivation, anthropomorphism, utilitarian performance expectancy, effort expectancy, and emotion). Moreover, they argued that the multi-stage appraisal was certified to be effective in the context of AI robotic devices used in hospitality services (Gursoy et al., 2019; Lin et al., 2019).

**Table 1:** Researches on AI technology acceptances.

**Types of AI**	**Key stimulus events**	**Influence mechanism**	**Behavioral intention/behavior**	**References**
Smart home services	Mobility, inter-operability, security, risk, and trust	Attitude, subjective norm, and perceived behavioral control	Intention to use	Yang et al., 2017
Smart home technology	Compatibility, privacy	Attitude	Intention to use	Shin et al., 2018
	Compatibility, liability, result demonstrability, visibility	Perceived usefulness, perceived ease of use	Behavioral intention	Hubert et al., 2019
	Compatibility, trialability, observability	Perceived usefulness, perceived ease of use	Intention to use	Nikou, 2019
	Optimism, innovativeness, discomfort, insecurity	Perceived risk, trust, engagement	Intentions to adopt	Mulcahy et al., 2019
Smart speakers	Product/platform-related variables	Perceived benefit, perceived risk, perceived value	adoption intention	Park et al., 2018
Voice assistant	Subjective norm	Perceived usefulness, perceived ease of use, attitude, engagement	Loyalty	Moriuchi, 2019
	Enjoyment, social presence, social cognition, privacy	Trust, attitude	Intentions to use	Pitardi and Marriott, 2021
Intelligent personal assistants	Task/social/physical attraction, security	Parasocial relationship, satisfaction	continuance intention	Han et al., 2018
AI artifacts	Sensing/thought/action autonomy	Competence/warmth perception	continuance usage intention	Hu et al., 2021
Chatbot	Mind perception	Closeness	Intention to use	Lee et al., 2020
Smart wearable devices	Service and system quality	Confirmation, perceived ease of use, perceived usefulness, perceived enjoyment, satisfaction	continuance intention to use	Park, 2020
Service robots	Anthropomorphism	Animacy, intelligence, safety, ease of use, usefulness, rapport, satisfaction	intention to use	Blut et al., 2021
AI devices	Social influence, hedonic motivation, anthropomorphism	Performance expectancy, effort expectancy, emotion	Willingness to accept/objection	Gursoy et al., 2019

However, AI devices can be classified into two types (social-oriented vs. task-oriented) (Van Doorn et al., 2017). Social-oriented AI devices can engage customers more effectively on a social level by expressing self-identity information through relationship development (Bickmore and Cassell, 2001). By strengthening the devices' empathy, AI robots can build up social and emotional connections with their human partners more effectively (Zhou et al., 2020). On the other hand, Task-oriented AI devices are created to help customers to complete tasks and achieve functional goals (e.g., answering routine questions). They are more straightforward and goal-oriented, and the functional values such as speed, accuracy, and efficiency are emphasized.

Current studies mainly focused on social-oriented AI devices, while little attention was paid to task-oriented AI devices. Sometimes, customers only want to complete a task (e.g., fix a problem or look for an answer) instantly, and they care more about time and accuracy. Although AIDUA model is a widely accepted model for AI device acceptance, it is constructed by hedonic value, anthropomorphism, and social influence, which can be seen as social-oriented features. In this regard, current theoretical models may not fully delineate the psychological path-way that a customer chooses to use task-oriented AI devices and how the antecedents influence tourists' intention to switch from AI devices to human staff when they interact with AI devices.

In addition, regarding the behavioral intention of consumers, existing studies mainly studied the willingness/objection to use AI devices. Generally, customers always face two options (AI vs. human) when they seek service assistance. However, AI servers are always set as default. Only when customers request human service, human staff is introduced. Therefore, switching intention may be the key process between the two options. Considering this point, it is important to study customers' intention to switch from task-oriented AI devices to human service.

### Research hypotheses based on a three-staged process

Following Lazarus's (1991b) cognition-motivation-emotion framework, and the multi-level cognitive appraisal process of the stimulus (Breitsohl and Garrod, 2016; Lv and McCabe, 2020), a three-stage process was introduced in this paper to examine customers' switching intention from task-oriented AI devices to human service. It has been validated that individuals' actual behavior is determined by their intentions and willingness. For instance, hedonic motivation was found as the primary factor affecting the AI device adoption intention of a customer (Niemelä et al., 2017). Moreover, cognitive appraisal theory proposes that the behavior of individuals is also influenced by their emotions, led by the multi-level cognitive appraisal process. Novacek and Lazarus (1990) mentioned that cognition and motivation are the impact factors that influence emotion with regards to the will or connation. Since motivation is associated with the goal or the evaluation when an individual chooses to behave, it determines the emotion. Besides, cognition can assist individuals in understanding the environment where they live and further help them capture the significance of the encounters (Lazarus, 1991b). Therefore, cognition is the premise that directs motivation, emotion, and behavior. In short, if individuals cannot realize what is happening through cognition, they may not be able to integrate the behavior (Miller et al., 1960). According to the multi-level cognitive appraisal process of the stimulus, users will form a higher-level cognitive appraisal of new technology at the beginning, and then generate a self-experience based on the results of the primary appraisal, which further produces a tendentious behavioral choice (Breitsohl and Garrod, 2016). Therefore, customers with utilitarian goals will initially pay more attention to practical features, such as interaction convenience and task-technology fit, when they are motivated by utilitarian motivation. Then the emotion generated from the general evaluation (perceived competence) will subsequently impact their behaviors (Judd et al., 2005; Hu et al., 2021).

#### Primary appraisal

Motivation is considered as one of the most important antecedents of behavior (Dweck and Leggett, 1988). Motivations can be classified into two types: hedonic and utilitarian. With hedonic motivation, customers expect pleasure and enjoyment from the services provided by social-oriented AI devices (Gursoy et al., 2019). Regarding the AIDUA model, hedonic motivation was proved as an important factor in the primary appraisal of users' adoption of social-oriented AI devices. However, customers who aim to complete a task (such as consulting product parameters or querying information) have utilitarian motivation (Leftheriotis and Giannakos, 2014; Longoni and Cian, 2020). Although it has been proved that customers with hedonic motivation and utilitarian motivation behave differently, both hedonic motivation and utilitarian motivation are considered to be able to explain the generation of flow states and ultimately lead to behavioral responses in a virtual interactive environment (Jeon et al., 2017).

According to cognitive appraisal theory, customers will generate a comprehensive cognitive evaluation based on the cognition of the specific feature of an AI. Consumers with utilitarian motivation expect to complete tasks effectively with the help of task-oriented AI devices, so they are more likely to expect task-oriented AI devices respond accurately and efficiently (Leftheriotis and Giannakos, 2014). By evaluating a task-oriented AI device's features, such as immediacy, accuracy, and comprehensiveness, customers can perceive the competence of a task-oriented AI device (Gerow et al., 2013). Previous literature have confirmed the relationship between hedonic motivation and the perception of AI performance. Similarly, we proposed that utilitarian motivation is positively associated with the perceived competence of task-oriented AI devices.

In summary, the following hypotheses are proposed:

H1: Utilitarian motivation is positively associated with perceived competence.

Another key antecedent proposed in the first appraisal phase is interaction convenience. Interaction convenience is defined as the extent of convenience when a customer uses task-oriented AI devices. Convenience includes simple to use approach, concise interface, and smart access, and these features are the embodiment of the competence of task-oriented AI devices. Previous literature indicated that perceived convenience is an important predictor of customers' behavioral intention (Yoon and Kim, 2007; Chang et al., 2012). Customers may evaluate the competence of a task-oriented AI device by measuring the convenience of using it (Chang et al., 2012). The more convenient customers interact with a task-oriented AI device, the more competence they can perceive. Therefore, we proposed the following hypothesis:

H2: Interaction convenience is positively associated with perceived competence.

Task-Technology Fit (TTF) is defined as the degree to which technology assists an individual in performing their portfolio of tasks (Goodhue and Thompson, 1995). Task-related factors and technology-related factors are considered important dimensions of TTF (Goodhue and Thompson, 1995), so TTF well reflects the relevance between task-oriented AI devices and consumers' own tasks. Compared with social-oriented AI devices, from which customers seek emotional interaction, customers are more goal-oriented when they use task-oriented AI devices. Therefore, TTF is more applicable for using the task-oriented AI device since new technology is conducive to performance only when the functions provided by new technology are suitable for users' tasks according to TTF theory (Thompson, 1995; Chung et al., 2015). Therefore, we propose that if customers perceive a high level of TTF, they are more likely to give a higher rate on the competence of the task-oriented AI devices:

H3: Task-technology fit is positively associated with perceived competence.

#### Secondary appraisal

In the secondary appraisal, customers may evaluate behavioral options and emotions toward the stimulus mentioned in the first appraisal (Lazarus, 1991a,b). Perceived competence and flow experience were introduced into the model. Perceived competence of the task-oriented AI device is considered as a comprehensive indicator that influences customers' behavioral intention, and flow experience is the emotional status generated. Perceived competence refers to the degree to which individuals perceive a task-oriented AI device's intelligence, efficacy, convenience, and efficiency (Fiske et al., 2002; Aaker et al., 2010; Hu et al., 2021). When customers interact with a task-oriented AI device that is simple to use, highly useful, and smart, customers are more likely to be completely absorbed in what they do. Previous literature named the state of immersion “flow state,” which describes the experience as fully immersed in a feeling of energized focus and acquiring enjoyment or full involvement through the process of engagement (Ellis et al., 1994). Flow experience was also defined as a state that is characterized by: a seamless sequence of responses facilitated by machine interactivity, intrinsic enjoyment, a loss of self-consciousness, and self-reinforcement (Hoffman and Novak, 1996). Therefore, we believe that a more competent task-oriented AI device is more likely to provide a flow experience for customers. Hence, we proposed that:

H4: Perceived competence is positively associated with flow experience.

#### Outcome stage

The outcome stage is the behavioral intention of customers to interact with task-oriented AI devices. Previous literature revealed a positive relationship between flow experience and customers' behavioral intention (Jeon et al., 2017). Similarly, when customers perceive that they may acquire a flow experience by interacting with the task-oriented AI devices, they may think that the task-oriented AI devices can solve their issues and help them reach their goals. Therefore, they may keep on using task-orient AI devices and decrease their switching intention from task-oriented AI devices to human service. We proposed that:

H5: Flow experience is negatively associated with switching intention.

**Figure 1:**
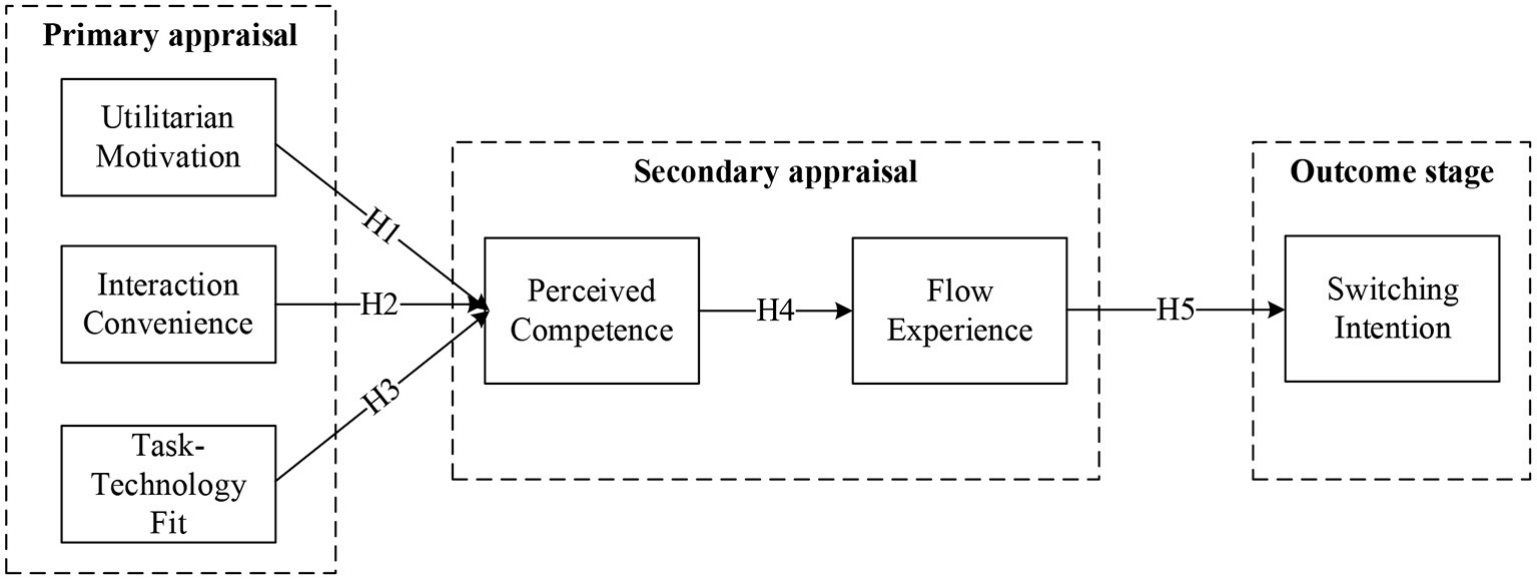
Theoretical model.

To sum up, the conceptual model proposed in this study is shown in Figure 1.

## Methodology

### Questionnaire development

A questionnaire with three sections was adopted in this study to examine the proposed theoretical model. The first section is a brief description of our study. We asked participants to recall an experience they had with task-oriented AI devices. An example of a typical task-oriented AI-based chatbot of Fliggy (www.fliggy.com) is presented to help participants recall their experiences. Fliggy, one of the largest online travel platforms in China, introduced an intelligent service chatbot, Yunxiaomi, to their website and mobile application in 2017 to provide customer services. If customers face any is issues they can directly contact Yunxiaomi on the product purchase interface and obtain assistance at any time. We provide an example of interacting with Yunxiaomi for reference (Appendix A in Supplementary material).

Section Theoretical background and hypotheses development covered 23 questions, which are used to measure the six constructs of the proposed model. Each construct is measured by 3–4 items using a 7-point Likert scale, with “1 = strongly disagree” and “7 = strongly agree”. According to the suggestion of James and Brett (1984), all of the items are derived from current literature to improve the content validity. The references for all items are indicated in Appendix B in Supplementary material.

Section Methodology consists of 5 questions to collect the demographic information of customers, including gender, age, education, occupation, and annual income. In order to avoid differences in the understanding of the questionnaires caused by language differences between Chinese and English. Three researchers participated in the Back-Translation. The first translator translated the original version of the questionnaire into Chinese. The second translator back-translated the translated version into English. The third translator compared the two versions and prepared the final version. At last, before issuing the formal questionnaire, a pre-test with 42 interviewees was conducted, and modification was conducted according to their feedback.

### Data collection

Data was collected by the Tencent Questionnaire platform (one of the most widely used online questionnaire-collection platforms in China, https://wj.qq.com). Tencent Questionnaire was utilized as the main source to collect online data by many leading researches and was certified reliable and valid (Tian et al., 2022; Zhu et al., 2022). Participants filled out questionnaires online in return for a small monetary reward, and three exclusion criteria were adopted for quality control: (1) when respondents answered the questionnaires, they were randomly embedded with dynamic verification codes (DVC) with time limits and attention check questions (ACQ). The responses who failed DVC/ACQ were excluded; (2) Responses with duplicate IPs (internet protocols) were excluded; (3) Responses with missing values were excluded. As a result, 419 valid responses were obtained in total. The effective response rate was 95.01%.

### Data analysis

In this study, we proposed a theoretical model to test the antecedents that influence customers' switching intention from task-oriented AI devices to human staff. According to the suggestions of Anderson and Gerbing (1998), a two-step procedure (measurement model and structural model test) was adopted to examine the conceptual model. For the first step, a confirmatory factor analysis (CFA) was utilized to test the reliability and validity of the measurement model. For the second step, we analyzed the dataset *via* covariance-based structural equation modeling (CB-SEM). As the findings of Hair et al. (2016) and Sarstedt et al. (2016) showed, CB-SEM can provide smaller bias and a more accurate result than partial least squares structural equation modeling (PLS-SEM). Finally, multiple regressions were conducted to examine the proposed mediating effects.

## Results

### Demographic profile of respondents

As summarized in Table 2, 26.49% of the participants were male, and 73.51% of them were female. In addition, most of the participants were between 18 and 25 years old (67.78%), had a bachelor's degree (79.24%), worked as full-time students (45.82%), and had an annual income Under ¥ 29,999 (69.21%).

**Table 2:** Demographic profile of respondents.

**Items**	**Category**	**Frequency (n = 419)**	**Distribution (%)**	**Cumulative percentage (%)**
Gender	Male	111	26.49	26.50
	Female	308	73.51	100.00
Age	18–25	284	67.78	67.78
	26–35	94	22.43	90.21
	36–55	36	8.59	98.81
	56–65	3	0.72	99.52
	65 and above	2	0.48	100.00
Education background	Less than Bachelor's degree	63	15.04	15.04
	Bachelor's degree	332	79.24	94.27
	Master's degree and above	24	5.73	100.00
Occupation	Full-time student	192	45.82	45.82
	Production personnel	14	3.34	49.16
	Sales personnel	25	5.97	55.13
	Technicist	55	13.13	68.26
	Administrative staff	36	8.59	76.85
	Others	97	23.15	100.00
Annual income (¥)	Under 29,999	290	69.21	69.21
	30,000–59,999	56	13.37	82.58
	60,000–89,999	35	8.35	90.93
	90,000–119,999	24	5.73	96.66
	120,000 and above	14	3.34	100.00

### Measurement model assessment

A confirmatory factor analysis (CFA) was conducted. Firstly, the absolute value of skewness and kurtosis are all <2 (Appendix B in Supplementary material), indicating good normality of the measurement items (Hair et al., 1998; Asghar and Saleh, 2012). Then we conducted reliability analyses of all constructs and the whole scale. As shown in Table 3, The Cronbach's alpha of the set of the scale was 0.925, and the Cronbach's alpha values of six constructs were all greater than the cut-off value of 0.70 (Nunnally and Bernstein, 1978), ranging from 0.723 (SI) to 0.943 (UM). Before estimating the factor loading of all items, we conducted a Kaiser-Meyer-Olkin (KMO) and Bartlett's sphericity tests. The KMO was 0.941, which was greater than the recommended value 0.80, indicating that our dataset was well suited for factor analysis (Nunnally and Bernstein, 1978). The results of factor analysis showed that factor loading of the utilized items were all above 0.7 except SI1 (0.694), and they were significant at 0.05 level, indicating a strong internal consistency (Fornell and Larcker, 1981). Last, the composite reliability (CR) scores of constructs were all above 0.7 (Gefen et al., 2000), showing good reliability.

**Table 3:** Measurement scale properties.

**Construct**	**Items no**.	**Factor loading**	**C. A**.	**AVE**	**CR**
Utilitarian motivation	UM1	0.930	0.943	0.854	0.959
	UM2	0.935			
	UM3	0.913			
	UM4	0.918			
Interaction convenience	IC1	0.889	0.917	0.805	0.925
	IC2	0.902			
	IC3	0.900			
	IC4	0.889			
Task-technology fit	TTF1	0.897	0.922	0.812	0.945
	TTF2	0.911			
	TTF3	0.915			
	TTF4	0.882			
Perceived competence	PC1	0.847	0.904	0.773	0.911
	PC2	0.882			
	PC3	0.908			
	PC4	0.894			
Flow experience	FE1	0.936	0.937	0.844	0.942
	FE2	0.919			
	FE3	0.900			
	FE4	0.916			
Switching intention	SI1	0.694	0.723	0.652	0.848
	SI3	0.884			
	SI4	0.833			

*C. A., Cronbach's alpha; AVE, average variance extracted; CR, composite reliability*.

Then the validity was examined. The average variance extracted (AVE) values for all constructs were greater than the recommended value of 0.5, suggesting an acceptable convergent validity (Bagozzi and Yi, 1998). Next, a correlation analysis was used to examine the discriminant validity of constructs, as the suggestion by Fornell and Larcker (1981). As shown in Table 4, the square root of AVEs (reported in the diagonal of the correlation matrix) of all constructs were greater than the correlation coefficients between themselves and other constructs, indicating acceptable discriminant validity.

**Table 4:** Discriminant validity analysis.

**Construct**	**UM**	**IC**	**TTF**	**PC**	**FE**	**SI**
UM	**0.924**					
IC	0.593	**0.897**				
TTF	0.539	0.432	**0.901**			
PC	0.674	0.748	0.510	**0.879**		
FE	0.532	0.541	0.443	0.739	**0.918**	
SI	−0.367	−0.227	−0.229	−0.378	−0.456	**0.808**

*UM, utilitarian motivation; IC, interaction convenience; TTF, task-technology fit; PC, perceived competence; FE, flow experience; SI, switching intention.*

*Number in bold on the diagonal indicate the square root of average variance extracted*.

### Structural model assessment

In this section, we utilized a CB-SEM to examine our theoretical model and estimated the relationships between the constructs. The chi-squared value was 521.880 with 220 degrees of freedom. All fit values indices were greater than the recommended ones (Table 5), suggesting that the structural model had a good fit (Bagozzi and Yi, 1998; Gefen et al., 2000).

**Table 5:** Recommended and actual values of fit indices.

**Fit indices**	**CMIN/DF**	**GFI**	**AGFI**	**PGFI**	**CFI**	**NFI**	**PNFI**	**IFI**	**TLI (NNFI)**	**RMSEA**
Recommended value	<3	>0.90	>0.80	>0.50	>0.90	>0.90	>0.50	>0.90	>0.90	<0.08
Actual value	2.372	0.905	0.881	0.722	0.964	0.940	0.817	0.964	0.959	0.057

*CMIN/DF, ratio between chi-squared and degrees of freedom; GFI, goodness of fit index; AGFI, adjusted goodness of fit index; PGFI, parsimony goodness of fit index; CFI, comparative fit index; NFI, normed fit index; PNFI, parsimony normed fit index; IFI, incremental fit index; TLI (NNFI), Tucker-Lewis index (non-normed fit index); RMSEA, root mean square error of approximation*.

**Figure 2:**
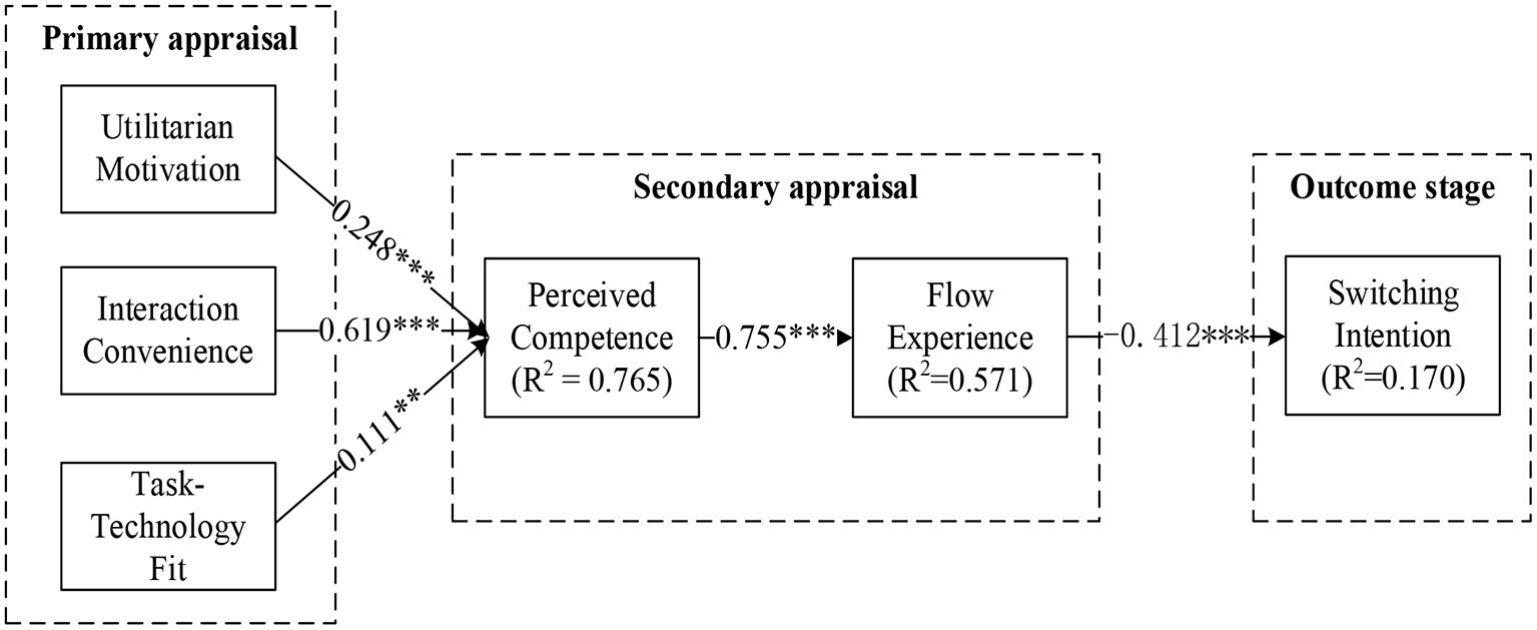
The results of the proposed theoretical model. *p < 0.1, **p < 0.05, ***p < 0.01.

The results of direct effects are shown in Table 6 and Figure 2. Hypotheses H1–5 were supported, and none of the standard errors (S.E.) of estimated parameters are negative, which indicates that the results of the path analysis were valid (Bagozzi and Yi, 1998). More specifically, in the first appraisal, utilitarian motivation (β = 0.248, *P* < 0.01), interaction convenience (β = 0.619, *P* < 0.01), and Task Technology Fit (β = 0.111, *P* < 0.05) had a significant positive effect on perceived competence separately, indicating H1–H3 were confirmed. Moreover, in the second appraisal, perceived competence was significantly and positively associated with flow experience (β = 0.755, *P* < 0.01), and H4 was supported. In addition, flow experience was significantly and positively related to switching intention (β = −0.412, *P* < 0.01), and H5 was supported.

**Table 6:** Direct path analysis.

**The hypotheses**	**Path coefficient**	**S.E**.	**Support**
H1: Utilitarian motivation → perceived competence	0.248^***^	0.049	Yes
H2: Interaction convenience → perceived competence	0.619^***^	0.046	Yes
H3: Task-technology fit → perceived competence	0.111^**^	0.046	Yes
H4: Perceived competence → flow experience	0.755^***^	0.049	Yes
H5: Flow experience → switching intention	−0.412^***^	0.034	Yes

*S.E., standard error.*

^*^p <0.1;

** *p <0.05;*

*** *p <0.01*.

Figure 2 shows the results of structural equation modeling and the test results of a significant portion of the variance in each construct. The R^2^ values of perceived competence, flow experience, and switching intention reached 0.765, 0.571, and 0.170, respectively, which explain 76.5%, 57.1%, and 17.0% of the variance in the corresponding constructs.

To further test the serial mediating effect of perceived competence and flow experience, the bootstrapping method was adopted for estimation with utilitarian motivation, interaction convenience, and TTF introduced as the independent variables, perceived competence and flow experience as the mediators, and switching intention as the dependent variable separately. The results (Table 7) revealed path “UM → PC → FE → SI” was significant [indirect effect = −0.077, CI = (−0.144, −0.036), not including 0], path “IC → PC → FE → SI” was significant [indirect effect = −0.193, CI = (−0.325, −0.122), not including 0] and path “TTF → PC → FE → SI” was significant [indirect effect = −0.034, CI = (−0.075, −0.01), not including 0]. As a result, PC and FE played the serial mediating role between UM/IC/TTF and SI.

**Table 7:** Parameter estimates in the structural model.

**Regression paths**	**Standardized indirect effects**	**SE**	**95% CI**		***p***
			**LLCI**	**ULCI**	
UM → PC → FE → SI	−0.077	0.028	−0.144	−0.036	<0.01
IC → PC → FE → SI	−0.193	0.047	−0.325	−0.122	<0.01
TTF → PC → FE → SI	−0.034	0.017	−0.075	−0.01	<0.01

*UM, utilitarian motivation; IC, interaction convenience; PC, perceived competence; TTF, task-technology fit; FE, flow experience; SI, switching intention; n.s., not significant; BC, bias-corrected*.

## Discussion and conclusion

Focusing on task-oriented AI devices, this study proposed a three-stage model to appraise customers' switching intention in using task-oriented AI devices. The measurement model and structural model were tested by CFA and CB-SEM through Amos 5.0. The results showed all eight hypotheses are supported.

The findings revealed that utilitarian motivation, interaction convenience, and task-technology fit are positive predictors of perceived competence (H1, H2 and H3), which means customers will evaluate these factors in the first stage when they get in touch with the task-oriented AI devices. Among these, interaction has the largest coefficient, indicating customers pay more attention to simplicity. When customers are task-oriented, their goal is to complete the task. Therefore, time and accuracy are more related. Convenience can save customers' time, and an inconvenient AI device may generate a sense of difficulty and time-consuming for customers to engage with. This is also the reason why customers use AI technology, since AI is regarded as time-saving. Moreover, TTF is another factor that influences customers' evaluation of task-oriented AI devices' competence. TTF is related to accuracy. When a task-oriented AI has better task-technology fit, it is regarded as the one that can provide better function. In addition, it is worth to be noted that the proposed T-AIA model introduced utilitarian motivation as a predictor, which is more suitable for the acceptance context of task-oriented AI devices. For AI devices that provide entertainment services (e.g., AI voice assistant), customers expect to obtain fun and pleasure from interaction (Gursoy et al., 2019). Therefore, hedonic motivation is considered an important antecedent for the acceptance of social-oriented AI devices (Sam et al., 2019). However, when customers are task-oriented, utilitarian motivation is more accurate in describing customers' internal stimulus (Lowe et al., 2013).

Moreover, we found a positive relationship between perceived competence with flow experience, and H4 was confirmed. In the secondary appraisal, customers will generate a comprehensive evaluation of using task-oriented AI devices, which further influence their emotional state. As the results showed, when customers perceive a higher level of AI competence, they think they are more likely to have a flow experience. Furthermore, the serial mediation of perceived competence and flow experience is verified. The findings showed that customers would generate a second appraisal of emotional status after they evaluate the stimulus in the first stage, and this process is consistent with the framework of Lazarus (1991b). However, different from Lazarus (1991b) and Gursoy et al. (2019), the findings showed that customers would generate a comprehensive evaluation of task-oriented AI devices, and further generate an emotional status based on the competence evaluation.

Finally, customers' flow experience was negatively associated with customers' switching intention; H5 was confirmed. Customers are more willing to switch to human staff when they encounter an unpleasant experience with task-oriented AI devices. For instance, when task-oriented AI devices can not answer the question correctly or solve customers' issues efficiently, they will cause anxiety and impatient for customers. In this situation, customers can not acquire full involvement, which may drive them to switch to human service as a result.

### Theoretical contribution

The present study makes important theoretical contributions to the literature. First, responding to the call for more study on task-and social-oriented AI devices (Chattaraman et al., 2019), this paper focused on the antecedents and psychological mechanism of customers' switching intention from task-oriented AI to human staff. Previous studies did not examine different types of AI devices separately (task-oriented AI vs. social-oriented AI) when they created the theoretical model of AI acceptance for customers (Lv et al., 2020; Chi et al., 2022). Considering that many customers are task-oriented, the T-AIA model proposed in this study provides a new perspective in explaining the use of task-oriented AI devices. This study segments AI devices by providing a more accurate model for explaining the underlying psychological mechanism of the interaction between customers and task-oriented AI devices. This study extends the knowledge on the use of AI technology by centering on task-oriented AI devices.

Second, the critical antecedents of customers' willingness to continuously use task-oriented AI devices are identified. The results show that utilitarian motivation, interaction convenience, task-technology fit, perceived competence, and flow experience are the most important determinants of the customers' intention to switch from task-oriented AI devices to humans. The findings were consistent with previous studies that utilitarian motivation is a critical factor that stimulates customers to adopt new technology (McLean and Osei-Frimpong, 2019; Stein and Ramaseshan, 2020; Vitezić and Perić, 2021). For instance, Stein and Ramaseshan (2020) found customers (hedonic vs. utilitarian motivation orientation) weighted differently on the overall customer experience when they evaluated the real-time touchpoint on a mobile app. However, few studies explored the underlying mechanism of how utilitarian motivation influences customers' switching intention. As evidenced in this paper, utilitarian motivation is identified as an important driver for the use of task-oriented AI devices, which is different from the acceptance model proposed by Chi et al. (2022). Moreover, interaction convenience and task-technology fit were the main consideration for customers in the task-oriented setting, compared with social influence and anthropomorphism in the use of social-oriented AI devices. Because when customers are goal-oriented, they place more attention on task solutions, which are associated with speed, accuracy, and efficiency. Customers may turn to human service when they run into an issue with the task-oriented AI devices instantly since they are impatient and do not want to wait. Regarding this point, interaction convenience and task-technology fit are more suitable to delineate the interaction process between customers and task-oriented AI devices.

Third, this study revealed the underlying mechanism of how the appraisal of task-oriented AI devices influences the switching intention of customers from using task-oriented AI devices to seeking assistance from human service. The results showed that perceived competence and flow experience played serial mediating roles, which means before customers generate the switching intention from task-oriented AI devices to human service, they will appraise perceived competence and flow experience following the evaluation of utilitarian motivation and interaction convenience, task-technology-fit in the first stage. Although the theoretical model was constructed on the basis of multi-level cognitive appraisal theory (Breitsohl and Garrod, 2016), this study extended the application of Lazarus's (1991b) cognition-motivation-emotion framework and the multi-level cognitive appraisal theory (Breitsohl and Garrod, 2016) by introducing a more accurate model to explain the use of task-oriented devices.

### Managerial implications

AI devices have been widely used by enterprises to provide services to customers instead of human staff. However, some customers still prefer to interact with human stuff rather than AI devices when accessing services (Lu et al., 2019). Therefore, it is critical for enterprises to understand the influence mechanism of internal and external stimuli on customers' adoption of using AI devices for services. By proposing the T-AIA model for task-oriented AI devices, the managerial implications of this paper are as follows:

First, utilitarian motivation, interaction convenience, and task-technology fit are important antecedents to reduce customers' switching intention from task-oriented AI devices to humans. Among them, interaction convenience is a critical one. Before customers become accustomed to using AI devices to replace human staff in obtaining services, product providers should spread the utilitarian and convenient advantages of the product more clearly. For example, if a company is trying to replace human customer service with task-oriented AI devices, then the company should spread the comparative advantages of task-oriented AI devices over human staff through public channels, such as “task-oriented AI devices can teach you to use coupons in less time” or “task-oriented AI devices can answer your queries 24 h a day”. Furthermore, enterprises should configure different types of AI (social-oriented or task-oriented) for customers with different service value (hedonistic or utilitarian) orientations in different service contexts, which can effectively enhance the continuous use of customers. At the same time, companies involving task-oriented AI devices need to be concerned about whether the AI devices they deploy are suitable for assisting customers with their intended tasks.

Second, perceived competence is a critical factor that can indirectly reduce customers' switching intention from AI to humans through the effect of flow experience. In the process of using AI services, if customers find that AI has the ability to complete tasks, which is matched with the initial evaluation, they will be more willing to use AI devices continuously to obtain services; otherwise, customers will switch to seek assistance from human staff. Therefore, business managers need to enhance the service competence of the AI devices (e.g., adding an extra knowledge base that cascaded with the existing information in the AI system, strengthening the ability of semantic comprehension, and providing information more accurately than human staff for the same question) and reduce human errors (e.g., identifying customers' demands mistakenly), enabling customers to immerse themselves in the interaction with AI.

Third, the present study proposed a theoretical model named T-AIA model, which is more suitable for business managers to understand customer behavior in using task-oriented AI devices. Interaction convenience and task-technology fit were identified as the critical antecedents of customers' switching intention from task-oriented AI devices to humans. Therefore, business managers who introduce task-oriented AI devices to serve customers should pay more attention to improving these two features, which can further lead customers to a state of flow. By engaging customers with efficient task-oriented AI devices, a business can decrease customers' switching intention from AI devices to human staff.

### Limitations and future research directions

Our research has the following limitations. First, the sample of this study was collected from the same country. Therefore, whether the findings can be applied to other countries need further examination. Previous studies showed that culture might impose influences on the technology acceptance behavior of users (Venkatesh and Zhang, 2010). Therefore, future studies can examine this model in a different culture. Second, our model only focused on task-oriented AI devices; future research could improve the generality by taking task- and social-oriented AI technology into consideration. Furthermore, this study proposed the T-AIA model to examine tourists' acceptance of AI service devices. However, this does not mean that the T-AIA framework is the only framework that is appropriate to investigate the task-oriented AI device acceptance for tourists. Other factors from a different perspective can also be considered in the future.

## Data availability statement

The raw data supporting the conclusions of this article will be made available by the authors, without undue reservation.

## Author contributions

YY and TL: conception and design of the study. YY organized the data. JL performed the analysis and review. TL wrote the first draft of the manuscript. All authors contributed to manuscript revision, read, and approved the submitted version.

## Conflict of interest

The authors declare that the research was conducted in the absence of any commercial or financial relationships that could be construed as a potential conflict of interest.

## Publisher's note

All claims expressed in this article are solely those of the authors and do not necessarily represent those of their affiliated organizations, or those of the publisher, the editors and the reviewers. Any product that may be evaluated in this article, or claim that may be made by its manufacturer, is not guaranteed or endorsed by the publisher.
