# Supplementary material for: An empirical assessment of a modified artificially intelligent device use acceptance model—From the task-oriented perspective
Source: Front Psychol. 2022 Aug 9;13:975307. doi: 10.3389/fpsyg.2022.975307 (PMC9396124; doi:10.3389/fpsyg.2022.975307)
Supplement: Supplementary file 1 [file Table_1.DOCX]

Supplementary Material

# Appendix A

An example of interacting with a task-oriented AI device.


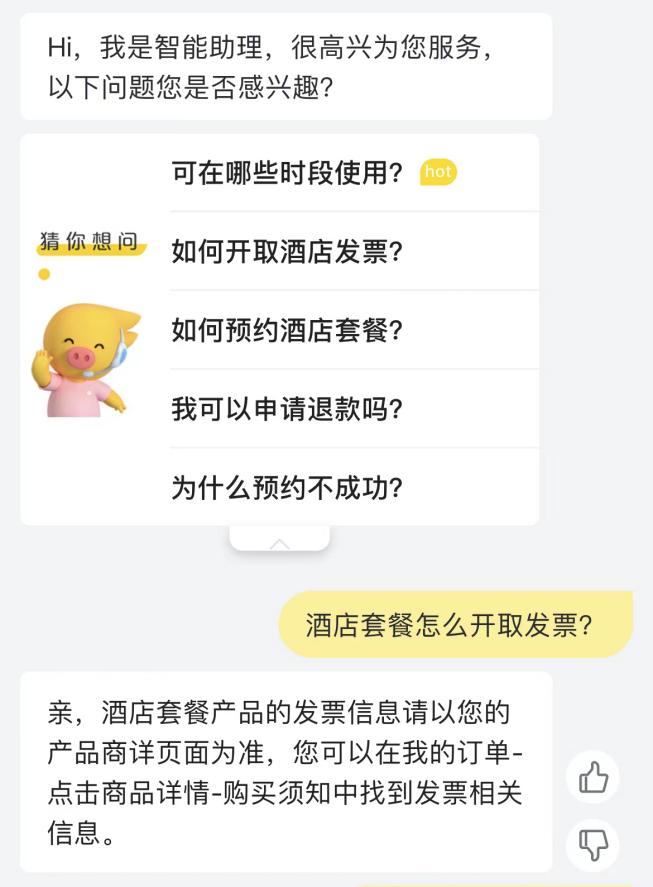


The dialogue in the picture is translated as follows:

[Task-oriented AI]

Hi, I am an intelligent assistant, glad to serve you. Are you interested in the following questions?

Supposing you want to ask...

- When can you use it?
- How to issue hotel invoices？
- How to book a hotel package？
- Can I apply for a refund?
- Why did your appointment fail?

*[Customer]*

*How to issue the invoice of a hotel package？*

[Task-oriented AI]

Dear, please refer to your product details page for invoice information of hotel package products. You can find relevant invoice information in "My Order" - "Click product details" - "Purchase Instructions".

# Appendix B

Measurement items statistics.

| **Construct** | **Item NO.** | **Items** | **Means** | **SD** | **Skewness** | **Kurosis** | **Reference** |
| --- | --- | --- | --- | --- | --- | --- | --- |
|  |  | Using task-oriented AI (e.g. AI chatbot) for your task is... |  |  |  |  | Cai, Liu, Zhao, & Li (2020) |
| Utilitarian motivation | UM1 | Effective. | 4.27 | 1.485 | -0.158 | -0.272 |  |
|  | UM2 | Helpful. | 4.45 | 1.459 | -0.263 | -0.209 |  |
|  | UM3 | Functional. | 4.24 | 1.532 | -0.226 | -0.472 |  |
|  | UM4 | Practical. | 4.25 | 1.545 | -0.147 | -0.463 |  |
| Interaction Convenience | IC1 | I can use task-oriented AI at any time for services. | 4.40 | 1.800 | -0.323 | -0.804 | Chang, Yan, & Tseng (2012) |
|  | IC2 | I can use task-oriented AI at any place for services. | 4.37 | 1.832 | -0.235 | -0.851 |  |
|  | IC3 | The use of task-oriented AI is convenient for me to conduct services. | 4.50 | 1.661 | -0.377 | -0.476 |  |
|  | IC4 | I feel that using chatbots is convenient for me for services. | 4.38 | 1.668 | -0.381 | -0.495 |  |
|  |  | In helping me to perform the assigned tasks, AI-based chatbot were very... |  |  |  |  | Chung, Lee, & Choi (2015) |
| Task-Technology Fit | TTF1 | Adequate | 4.10 | 1.500 | -0.129 | -0.498 |  |
|  | TTF2 | Compatible with task | 4.17 | 1.423 | -0.21 | -0.369 |  |
|  | TTF3 | Helpful | 4.29 | 1.427 | -0.172 | -0.387 |  |
|  | TTF4 | Made the task very easy | 4.18 | 1.478 | -0.127 | -0.425 |  |
|  |  | Task-oriented AI (e.g. AI chatbot) is... |  |  |  |  | Hu, et al. (2021) |
| Perceived Competence | PC1 | Intelligent. | 3.77 | 1.800 | 0.059 | -0.957 |  |
|  | PC2 | Skillful. | 4.18 | 1.720 | -0.165 | -0.752 |  |
|  | PC3 | Capable. | 3.88 | 1.713 | -0.008 | -0.763 |  |
|  | PC4 | Effective. | 4.18 | 1.598 | -0.054 | -0.609 |  |
| Flow Experience | FE1 | When I was interacting with task-oriented AI (e.g. AI chatbot), I felt totally captivated. | 3.08 | 1.734 | 0.494 | -0.698 | Jeon, Lee, & Jeong, (2017) |
|  | FE2 | When I was interacting with task-oriented AI (e.g. AI chatbot), time seemed to pass very quickly. | 3.33 | 1.822 | 0.367 | -0.854 |  |
|  | FE3 | When I obtained customer service from task-oriented AI (e.g. AI chatbot), nothing seemed to matter to me. | 3.26 | 1.790 | 0.359 | -0.928 |  |
|  | FE4 | When I obtained customer service from task-oriented AI (e.g. AI chatbot), I was full involved. | 3.35 | 1.708 | 0.294 | -0.791 |  |
| Switch Intention | SI1 | I prefer to consider task-oriented AI (e.g. AI chatbot) as my primary customer services provider. | 4.52 | 1.550 | -0.012 | -0.684 | Wu, Vassileva, & Zhao. (2017) |
|  | SI2 | I am determined to switch to obtain assistance from human service. | 4.95 | 1.402 | -0.355 | -0.402 |  |
|  | SI3 | I think my switch behavior from AI (e.g. AI chatbot) to human will happen in the recent months. | 4.81 | 1.420 | -0.299 | -0.153 |  |
